# Predictors of Seizure Freedom in Patients Undergoing Surgery for Central Nervous System Infection-Related Epilepsy: A Systematic Review and Meta-Analysis

**DOI:** 10.3389/fneur.2021.668439

**Published:** 2021-08-18

**Authors:** Zhi Hou, Qing-Tian Duan, Yan-Yan Ke, Ning An, Hui Yang, Shi-Yong Liu, Chun-Qing Zhang

**Affiliations:** Department of Neurosurgery, Xinqiao Hospital, Army Medical University (Third Military Medical University), Chongqing, China

**Keywords:** central nervous system infection, epilepsy, surgery, seizure freedom, meta-analysis

## Abstract

**Objective:** Central nervous system infections (CNSIs), especially viral encephalitis and meningitis, are well-recognized causes of medically refractory epilepsy. Although surgery is an effective and durable intervention against these infections, the seizure control outcomes described in previous surgical series have been variable. Accordingly, it is not clear which variables are most valuable in predicting seizure control following surgery for CNSI. The aim of this meta-analysis was to identify the predictors of favorable surgical outcomes in CNSI-related epilepsy.

**Methods:** The PubMed, EMBASE, Cochrane Library, WANGFANG, VIP, CBM, and CNKI databases were searched for studies according to the inclusion criteria. Prognostic factors, surgical outcomes, and patient characteristics were extracted. Heterogeneity was detected by the I^2^ and Q statistics.

**Results:** Seventeen studies were included in our meta-analysis. Eight predictors of favorable outcomes (Engel Class I/II) were determined, including abnormal MRI findings, meningitis, temporal location only, regional ictal pattern, unilateral ictal pattern, older age at epilepsy, longer silent period, and longer time from infection, as follows: OR = 3.34 (95% CI 1.44–7.74), OR = 0.31 (95% CI 0.13–0.70), OR = 0.34 (95% CI 0.16–0.74), OR = 5.65 (95% CI 1.75–18.30), and OR = 9.53 (95% CI 2.36–38.48), respectively, and MD = 2.15 (95% CI 0.20–4.11), MD = 2.40 (95% CI 0.09–4.70), and MD = 8.49 (95% CI 1.50–15.48), respectively. A subgroup analysis found the following associations: regional and unilateral ictal patterns in viral encephalitis, a younger age at infection in parasitic encephalopathy, an older age at surgery, a longer time from onset, and a longer time from infection in unexplained meningitis. A sensitivity analysis restricted to studies that included each variable yielded robust results. Little evidence of publication bias was observed.

**Conclusions:** This meta-analysis suggests that abnormal MRI findings, meningitis, temporal location only, regional and unilateral ictal patterns, older age at epilepsy, longer silent period, and longer time from infection are predictive factors in patients with favorable surgical outcomes in CNSI-related epilepsy. In addition, different infective agents influenced the results in regional and unilateral ictal patterns in ictal electroencephalography, as well as the relationship between age at infection and surgery and the time from epilepsy onset and infection.

## Introduction

Central nervous system infections (CNSIs) are a frequent cause of acquired epilepsy worldwide (1, 2). Seven percent of CNSI survivors have a risk of developing late symptomatic epilepsy (3), especially in the first 5 years (4). Early (acute) symptomatic seizures present within 2 weeks after infection (5), whereas late unprovoked seizures occur months to years after CNSI. According to a small number of reports, infection eventually leads to epilepsy in 12–22% of affected children (6, 7) and 25% of affected adults (7). Various infectious agents can cause late symptomatic epilepsy, including viruses (38% of cases), neurocysticercosis (NCC) (34%), and tuberculosis (25%) (7). Epilepsy can also occur after CNSI involving other pathogens, such as bacteria, and other diseases, such as schistosomiasis.

Late symptomatic epilepsy is strongly related to adverse outcomes, including progressive cognitive and behavior impairment, pharmacoresistance and other epilepsy-associated morbidities, such as traumatic injury, depression, and sudden unexpected death (8–10). A multicenter French study reported that 40% of epilepsy cases that initiated after CNSI-developed drug resistance (11), although only 6% of pediatric and 8% of adult cases of intractable epilepsy were caused by “postnatal infections and other postnatal factors” (12). Despite the high rate of pharmacoresistance, surgery is a highly effective and durable intervention that can improve quality of life in patients with intractable epilepsy (13, 14). Moreover, early surgical intervention might release or reverse impaired psychosocial outcomes in patients with uncontrolled seizures during adolescence or adulthood (14–16).

The surgical outcomes of postinfectious epilepsy are encouraging in general; however, the prognostic factors that predict surgical outcomes remain unclear, although various potential risk factors have been identified. A recent case-control study showed that longer epilepsy durations and multiple lobe involvement predicted worse outcomes in NCC-related epilepsy (17), whereas another retrospective study reported that the age of occurrence of previous meningitis or a history of encephalitis contributed to outcomes after anterior temporal lobectomy. However, the predictors that have been related to surgical outcomes have varied among numerous studies. Therefore, we conducted the first meta-analysis to identify the real risk factors associated with seizure freedom and surgical outcomes in CNSI-related epilepsy.

## Method

A systematic literature review and meta-analysis was performed adhering to the recommendations of the Preferred Reporting Items for Systematic Reviews and Meta-Analyses (PRISMA) guidelines (Appendix 1) (18, 19).

### Literature Search

We performed a systematic search of the PubMed (MEDLINE), Embase, Cochrane Library, WANGFANG, VIP Database for China Science and Technology Journal (VIP), Chinese National Knowledge Infrastructure (CNKI), and Chinese Biomedical and Medical Database (CBM) databases from database inception to April 1, 2018. We applied the following terms as a search strategy: “surgery,” “central nervous system infections,” and “epilepsy” (the detailed strategy is presented in the Supplementary Material). No geography or language restrictions were imposed. When necessary, we contacted the study authors for additional information.

### Study Selection

Studies were included if they fulfilled the following criteria: (1) patients underwent surgery for postinfectious epilepsy; (2) surgical outcomes were documented in accordance with Engel's classification (20), the International League Against Epilepsy (ILAE) classification (21), or any other classifications match description based on the Engel (or ILAE) classification or the detailed outcomes of each patient were reported; (3) the predictive variables included in the study were associated with an Engel or ILAE classification or another acceptable classification match description similar to the Engel (or ILAE) classification for surgery for CNSI-related epilepsy; and (4) studies published as full articles, meeting abstracts with full data, theses, case series, or case reports. Studies were excluded if they failed to meet the inclusion criteria or provided incomplete or non-retrievable data. When the same population was used in more than one study, we selected the study that had the longest follow-up time.

Two independent authors screened the search results for potential inclusion according to the inclusion and exclusion criteria. All disputes were resolved by consensus.

### Data Extraction

Data were extracted systematically from the studies and compiled independently by two reviewers using standard electronic sheets and cross-checks to reach a consensus. In the case of any disagreement, a consensus was reached by discussion. Trial and patient characteristics were documented and included the name of the first author, year of publication, country, period, infective type, infective agent, gender, number of participants, mean age at surgery, disease course, standard outcome classification, and predictors. The predictors of interest were gender; age; side (dominat or non-dominant hemisphere and unilateral or bilateral), location (temporal and extratemporal locations), and number of lesions on magnetic resonance imaging (MRI)/computed tomography (CT); ictal and interictal electroencephalography (EEG); MRI structural abnormalities; type of disease (encephalitis or meningitis); choice of operation [anterior temporal lobectomy (ATL) or extratemporal cortisectomy (ETC)], and time from first seizure. A favorable outcome was defined as Engel I and II or ILAE OC1 and 2 (22). In five studies without an Engel or ILAE classification, only seizure freedom was regarded as a good prognosis for statistical purposes (23–27). If the lesion was located in both the temporal and the extratemporal locations, we considered it to have an extratemporal location. The time from first seizure/infection was defined as the time between surgery and the first seizure onset or infection. The silent period was defined as the time between acute infection and epilepsy onset.

The Newcastle-Ottawa Scale (NOS) was used to assess the quality of observational studies (28). The quality of case-control studies was also assessed according to three factors: the selection of cases and controls (0–4 stars), comparability of cases and controls (0–2 stars), and ascertainment of exposure (0–3 stars) (28).

### Outcome Measures

The main outcomes were MRI findings, the type of infection, the location, side and number of epileptic lesions, age of onset and silent period of epilepsy, and the time from infection to epilepsy.

### Statistical Analysis

We calculated individual and pooled odds ratios (ORs) with corresponding 95% confidence intervals (95% CIs) for each study. Heterogeneity in OR was estimated by the *I*^2^ statistic and *P*-values. A *P* < 0.05 or *I*^2^ > 50% (29) was defined as indicative of significant heterogeneity (30, 31), and in those instances, a random-effects model was used for the meta-analysis. Otherwise, we selected a fixed-effects model (32). Furthermore, we conducted a sensitivity analysis to test the robustness of the pooled results by omitting one study each in turn. A subgroup analysis was conducted to investigate the potential effects of different infective agents on outcomes. All analyses were performed using Review Manager Version 5.1.7 (provided by The Cochrane Collaboration, available at http://www.cc-ims.net/revman) and STATA Version 11.0 (StataCorp, College Station, TX). A *P*-value < 0.05 was considered to be significant, except where otherwise specified. Begg's correlation and Egger's regression tests were used to assess potential publication bias (33, 34). If publication bias was evident, the trim and fill method was applied to provide an adjusted summary OR that included potentially missing trials (35).

## Results

### Literature Search

**Figure 1:**
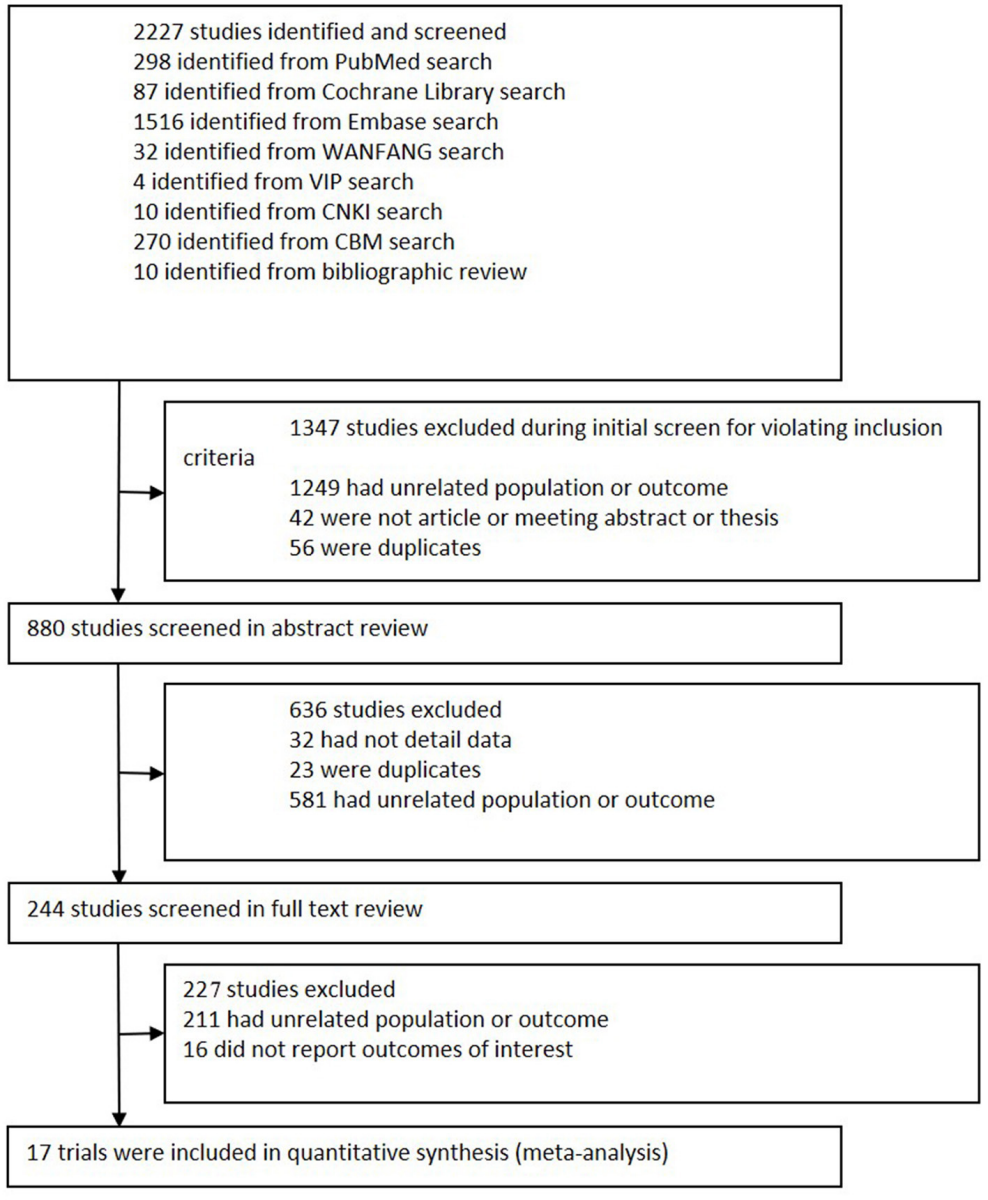
Flowchart of study selection. This flowchart shows the procedure used to select studies according to the inclusion criteria.

We initially retrieved 2,227 potential references from databases and bibliographies (Figure 1). Based on the inclusion criteria, 1,347 studies were excluded after title screening, and 636 trials were excluded after a review of the abstracts. A full-text review of the remaining 244 papers excluded 227 studies for the following reasons: 211 had an unrelated population or outcome, and 16 did not report outcomes of interest. Finally, 17 articles containing 390 patients who met all inclusion criteria were enrolled in the meta-analysis (17, 23–27, 36–46). All articles were subjected to intention-to-treat (ITT) analysis.

### Study Characteristics

The basic characteristics of the included studies are presented in Table 1. All the studies were published between 1984 and 2016. Of the included studies, seven were conducted in North America (four in the USA and three in Canada) (23–27, 39, 44), six in Asia (two in China, two in India, one in the State of Qatar, and one in South Korea) (36, 37, 40, 41, 43, 46), two in Europe (one in Spain and one in Austria) (38, 45), one in South America (Brazil) (17), and one in Oceania (Australia) (42). Regarding the writing language, 15 articles were written in English, and two were published in Chinese. For most studies, the mean age at the time of surgery ranged from 3 to 69 years old. The quality of observational studies was low to moderate, and only five studies received more than seven stars according to the NOS (Table 2).

**Table 1:** Characteristics of the included studies.

**Author publication year**	**Location**	**Period**	**Disease type**	**Infective agent**	**Gender (Male/Female)**	**No. of participants**	**Mean age at surgery (years)**
Liu et al. (41)	China	2007–2014	VE	NR	4/9	13	10 (3–23)
Wang et al. (46)	China	2006–2008	VE, PM/E	Unknown, EEBV, TB	19/10	29	18 (3–47)
Meguins et al. (17)	Brazil	2000–2013	PE	NCC	68/59	127	34.7 (21–68)
Chandra et al. (37)	India	1998–2006	M, PE, SE	NCC, TB, Unknown	18/10	28	20.4 (6–32)
Donaire et al. (38)	Spain	1995–2004	PE	NCC	11/6	17	38 (21–61)
Bashir et al. (36)	State of Qatar	1996–2000	SE	SM, BPM, NC	2/3	5	28 (11–45)
Trinka et al. (45)	Austria	1982–1998	VE		16/6	22	28.9 (5–52)
Trinka et al. (44)	Canada	1982–1999	VE	HSV, VZV, MV, MuV, CBV, PIV, Unknown	15/9	24	NR
Jay et al. (39)	Canada	NR	VE	HSV	0/2	2	7.3 (6–8.5)
Lancman et al. (25)	USA	1990–1993	CNSI	NR	NR	19	NR
Davies et al. (23)	USA	1986–1992	M	NR	8/5	13	22.3 (4–48)
Davies et al. (24)	USA	1986–1992	VE	NR	6/5	11	22.6 (8–37)
Leblanc et al. (26)	Canada	NR	PE	NCC	4/3	7	34.5 (21–55)
Mitchell et al. (27)	USA	1980–1984	PE	NCC	1/1	2	9.5 (9–10)
Rathore et al. (43)	India	2001–2010	PE	NCC	NR	15	25.7 (17–47)
O'Brien et al. (42)	Australia	1989–1996	M, E	NR	18/21	39	29 (12–69)
Lee et al. (40)	South Korea	1989–1995	CNSI	NR	9/9	18	28 (17–38)

*M, meningitis; E, encephalitis; VE, viral encephalitis; PE, parasitic encephalopathy; SE, subdural empyema; PM/E, purulent meningitis/ encephalitis; CNSI, central nervous system infection*.

*EEBV, epidemic encephalitis B virus; TB, tuberculosis; SM, Streptococcus melleri; BPM, Burkholderia pseudommallei; NC, negative culture; HSV, herpes simplex virus; VZV, varicella zoster virus; MV, measles virus; MuV, mumps virus; CBV, coxsackie B4 virus; PIV, parainfluenza virus; NCC, neurocysticercosis; NR, not reported; Mean Age at Surgery is expressed as mean (range)*.

**Table 2:** Quality of the included studies.

**Studies**	**Selection of case and controls**				Comparability of cases and controls ^*^	**Ascertainment of exposure**		**Non-response rate**	**Total quality scores**
	**Adequate definition of case**	**Representativeness of cases**	**Selection of control**	**Definition of control**		**Exposure assessment**	**Same method of ascertainment for cases and controls**		
Liu et al. (41)						^*^	^*^	^*^	3
Wang et al. (46)	^*^	^*^	^*^	^*^	^*^	^*^	^*^	^*^	8
Meguins et al. (17)						^*^	^*^	^*^	3
Chandra et al. (37)						^*^	^*^	^*^	3
Donaire et al. (38)						^*^	^*^	^*^	3
Bashir et al. (36)						^*^	^*^	^*^	3
Trinka et al. (45)						^*^	^*^	^*^	3
Trinka et al. (44)	^*^	^*^	^*^	^*^		^*^	^*^	^*^	7
Jay et al. (39)						^*^	^*^	^*^	3
Lancman et al. (25)	^*^	^*^	^*^	^*^	^*^	^*^	^*^	^*^	8
Davies et al. (23)						^*^	^*^	^*^	3
Davies et al. (24)						^*^	^*^	^*^	3
Leblanc et al. (26)						^*^	^*^	^*^	3
Mitchell et al. (27)						^*^	^*^	^*^	3
Rathore et al. (43)						^*^	^*^	^*^	3
O'Brien et al. (42)	^*^	^*^	^*^	^*^	^*^	^*^	^*^	^*^	8
Lee et al. (40)	^*^	^*^	^*^	^*^		^*^	^*^	^*^	7

* *Control for important factor or additional factor (A maximum of two stars can be awarded for Control for important factor or additional factor)*.

### Main Analysis

**Figure 2:**
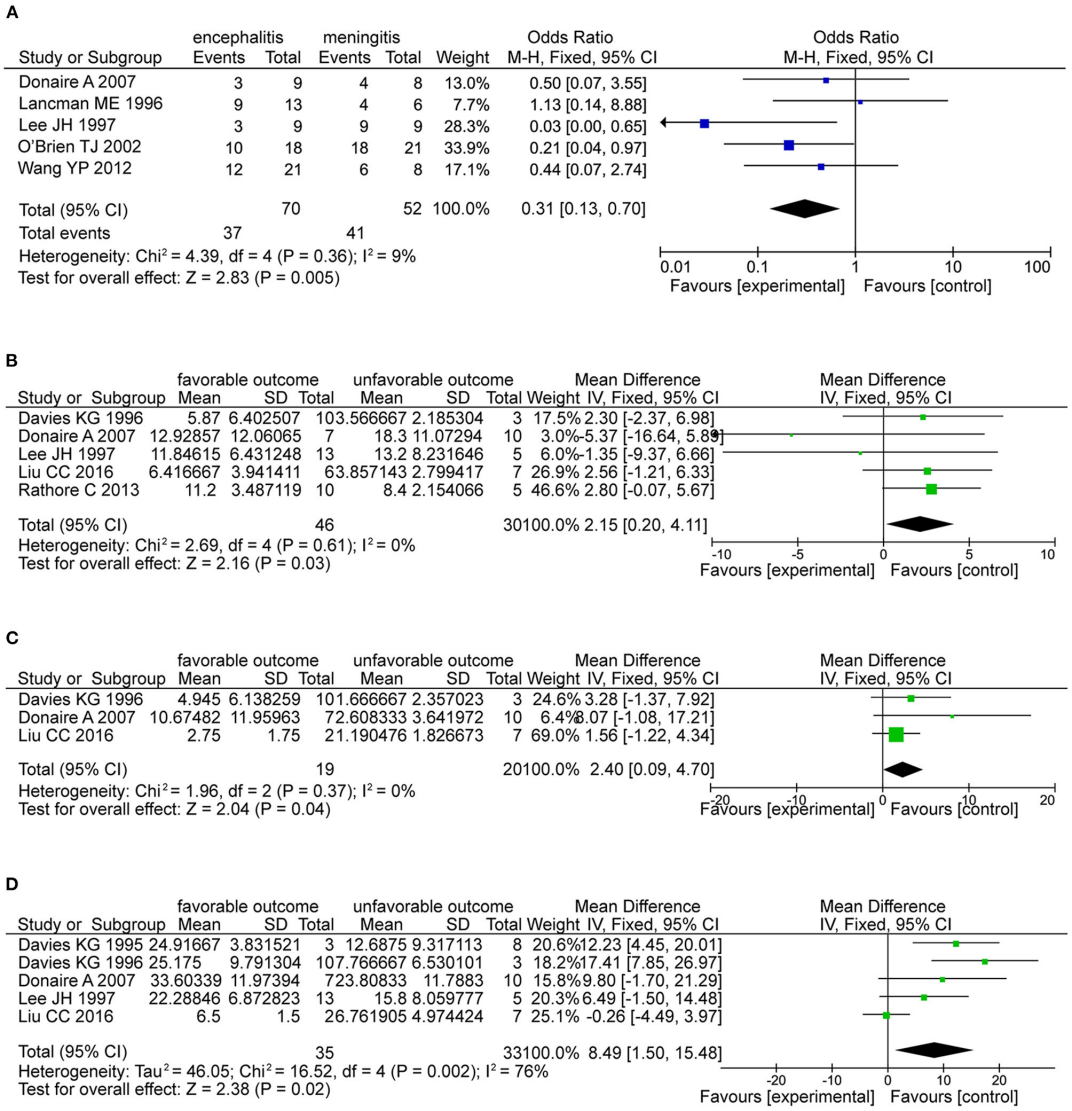
The factors associated with favorable outcomes. **(A)** The forest plot showed that meningitis was associated with favorable outcomes than encephalitis (OR 0.31, 95% CI 0.13–0.70, *P* = 0.005). **(B)** The forest plot showed older age at epilepsy (OR 2.15, 95% CI 0.20-4.11, *P* = 0.03) is associated with favorable outcomes. **(C)** Forest plot showed longer silent period (OR 2.40, 95% CI 0.09-4.70, *P* = 0.04) is associated with favorable outcomes. **(D)** Forest plot showed the time from infection (OR 8.49, 95% CI 1.50-15.48, *P* = 0.02) is associated with favorable outcomes.

**Figure 3:**
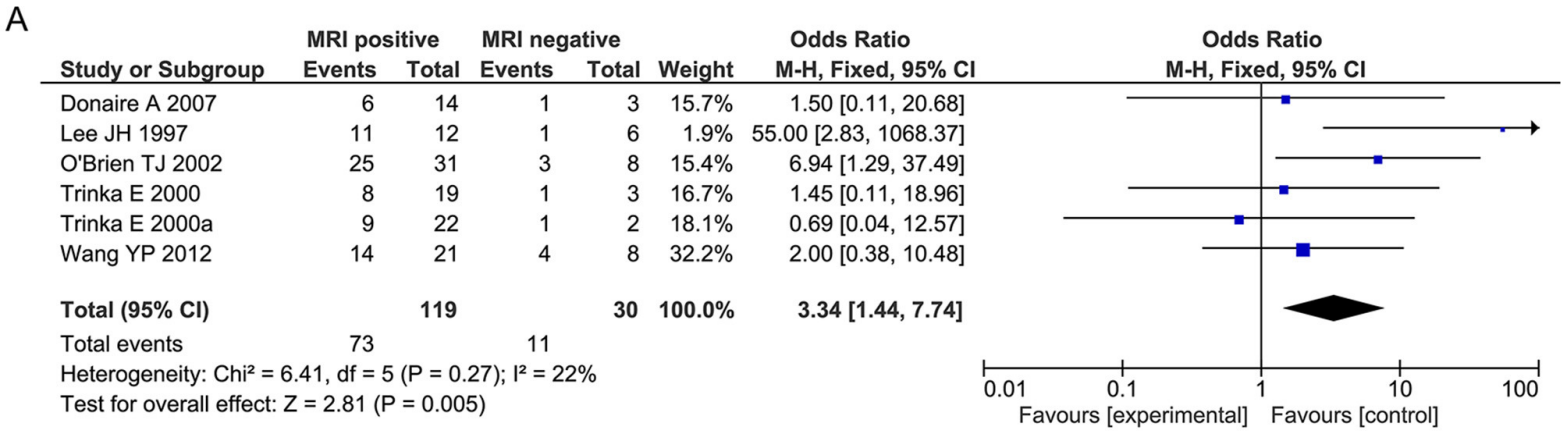
Meta-analysis for MRI findings. **(A)** Forest plot showed favorable outcomes were achieved in patients with positive MRI findings.

**Figure 4:**
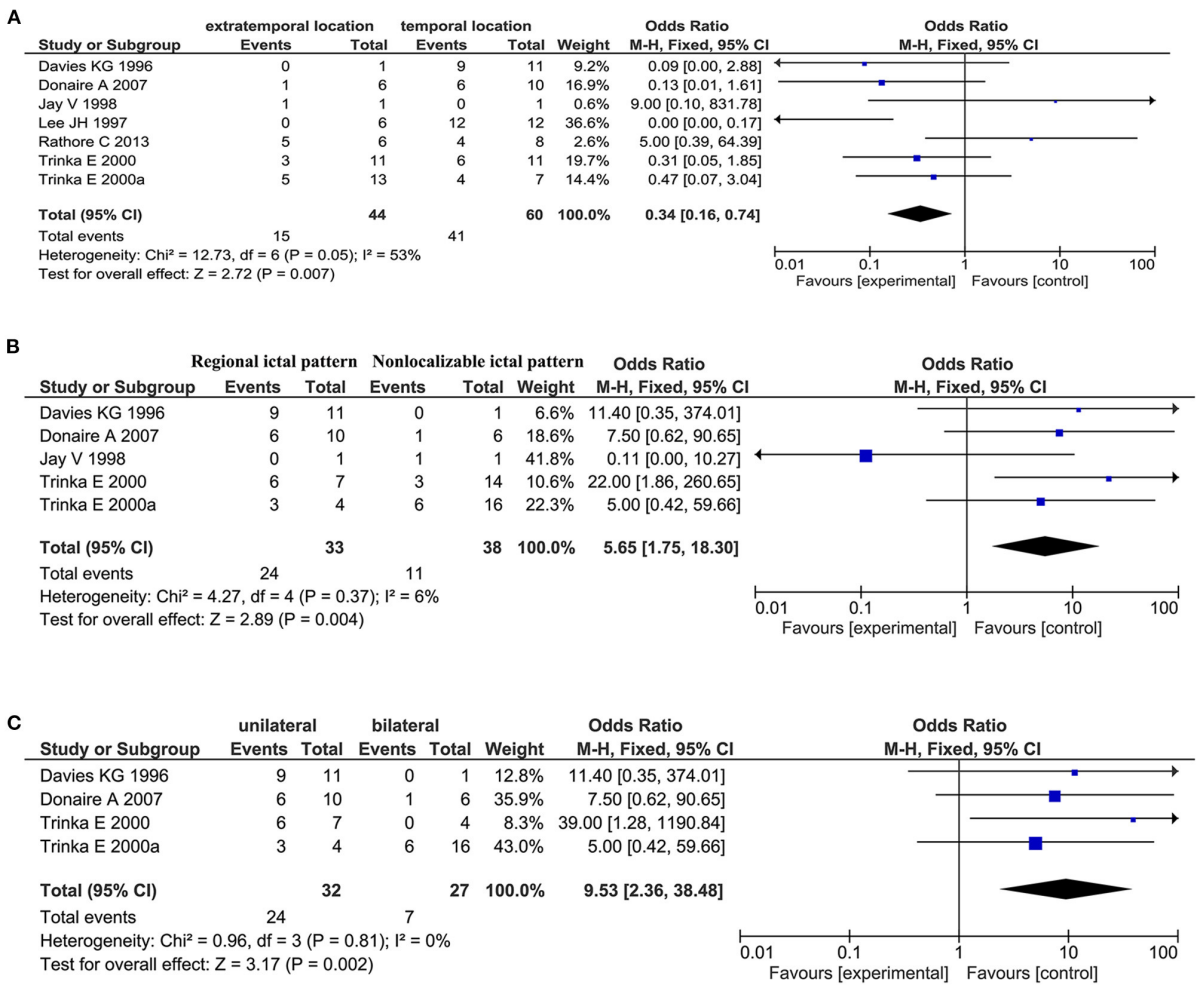
Meta-analysis of ictal EEG findings. **(A)** Forest plot showed a temporal location of only lesion was associated with favorable outcomes in patients. **(B)** Forest plot showed a regional ictal pattern of EEG findings was associated with favorable outcomes in patients. **(C)** Forest plot showed favorable outcomes were achieved in patients with unilateral ictal EEG findings.

Regarding general patient information, such as gender, disease type (encephalitis or meningitis), age at infection, onset, and surgery, time from seizure, silent period duration, and time from infection, meningitis was more frequently associated with favorable outcomes when compared with encephalitis (OR 0.31, 95% CI 0.13–0.70, *p* = 0.005, Figure 2A). Additionally, older age at surgery (MD = 2.15, 95% CI 0.20–4.11), longer time from seizure onset to surgery (MD = 2.40, 95% CI 0.09–4.70), and longer time from infection to surgery (MD = 8.49, 95% CI 1.50–15.48) were associated with better outcomes (Figures 2B–D). Regarding manifestations viewed on MRI, in CNSI epilepsy, an abnormal MRI finding was associated with a higher rate of favorable outcomes achieved in patients than a normal MRI result (OR 3.34, 95% CI 1.44–7.74, *p* = 0.005, Figure 3). With regard to ictal EEG features (temporal or extratemporal ictal pattern, regional or non-localizable ictal pattern, and unilateral or bilateral ictal pattern), we identified that temporal ictal pattern, regional ictal pattern, and unilateral ictal pattern were protective factors in the prognosis of postinfective epilepsy (extratemporal location, OR 0.34, 95% CI 0.16–0.74, *p* = 0.007; regional ictal pattern, OR 5.65, 95% CI 1.75–18.30, *p* = 0.004; unilateral ictal pattern, OR 9.53, 95% CI 2.36–38.48, *p* = 0.002, Figures 4A–C).

### Subgroup and Sensitivity Analyses

According to disease type, all the studies were classified into the following groups: viral encephalitis, parasitic encephalopathy, bacterial meningitis, and unexplained meningitis (the etiological agent of meningitis was not clearly identified). Among all variables of interest, single location of ictal EEG patterns or unilateral location of ictal EEG patterns are markers of a good prognosis in patients with history of viral encephalitis. This finding was consistent with the pooled results obtained by combining all groups, although no significant association was found in any of the other individual groups. The remaining primary outcomes failed to show any significant results among the four groups (Table 3).

**Table 3:** Subgroup analysis of primary outcomes.

**Items**	**Viral encephalitis**	***P-*valve**	**Parasitic encephalopathy**	***P-*valve**	**Bacterial meningitis**	***P-*valve**	**Unexplained meningitis**	***P-*valve**
**General information**								
Gender	1.41 (0.51, 3.91)	0.51	0.70 (0.15, 3.27)	0.65	0.33 (0.01, 12.82)	0.56	7.00 (0.29, 170.05)	0.23
Age at infection	−0.57 (−3.58, 2.44)	0.71	−13.44 (−21.22, −5.66)	0.0007			−0.97 (−2.73, 0.78)	0.27
Age at onset	2.56 (−1.21, 6.33)	0.18	2.30 (−0.48, 5.08)	0.10			2.30 (−2.37, 6.98)	0.33
Age at surgery	6.19 (−3.93, 16.30)	0.23	−2.83 (−10.01, 4.34)	0.44			16.43 (7.16, 25.71)	0.0005
Time from seizure	−0.99 (−4.91, 2.94)	0.62	−1.01 (−7.57, 5.55)	0.76			14.13 (7.03, 21.23)	<0.0001
Silent period	1.56 (−1.22, 4.34)	0.27	8.07 (−1.08, 17.21)	0.08			3.28 (−1.37, 7.92)	0.17
Time from infection	5.54 (−6.67, 17.75)	0.37	9.80 (−1.70, 21.29)	0.09			17.41 (7.85, 26.97)	0.0004
**MRI findings**								
Abnormal vs. Normal	1.06 (0.16, 7.05)	0.95	1.50 (0.11, 20.68)	0.76				
Dominant vs. Non-dominant	1.00 (0.05, 20.83)	1.00	1.41 (0.27, 7.31)	0.68	3.00 (0.08, 115.34)	0.56		
Extemporal vs. Temporal	3.00 (0.10, 88.13)	0.52	0.55 (0.06, 4.66)	0.58	0.21 (0.01, 5.05)	0.33	0.02 (0.00, 1.01)	0.05
Single vs. Multiple	0.50 (0.03, 7.45)	0.62	2.55 (0.51, 12.70)	0.25	3.21 (0.34, 30.04)	0.31		
Unilateral vs. Bilateral	4.34 (0.29, 65.01)	0.29	2.59 (0.38, 17.55)					
**Interictal EEG findings**								
Right vs. Left			1.74 (0.23, 13.05)	0.59				
Extemporal vs. Temporal			1.63 (0.31, 8.67)	0.56				
Single vs. Multiple			1.87 (0.37, 9.44)	0.45				
Unilateral vs. Bilateral			0.94 (0.03, 32.50)	0.97				
**Ictal EEG findings**								
Right vs. Left	0.07 (0.00, 1.65)	0.10	2.17 (0.38, 12.55)	0.39			1.00 (0.05, 20.83)	1.00
Extemporal vs. Temporal	0.52 (0.16, 1.70)	0.28	0.78 (0.19, 3.22)	0.73			0.09 (0.00, 2.88)	0.17
Single vs. Multiple EEG ictal onset	4.68 (1.13, 19.37)	0.03	7.50 (0.62, 90.65)	0.11			11.40 (0.35, 374.01)	0.17
Unilateral vs. Bilateral	10.48 (1.53, 71.75)	0.02	7.50 (0.62, 90.65)	0.11			11.40 (0.35, 374.01)	0.17
**Surgery**								
ATL vs. ETC	3.10 (0.71, 13.65)	0.13	0.93 (0.15, 5.67)	0.94			12.60 (0.39, 411.11)	0.15

*Silent period, the time between acute infection and the onset of epilepsy; ATL, anterior temporal lobectomy; ETC, extratemporal cortisectomy*.

Sensitivity analyses were conducted by sequentially omitting individual studies. The combined ORs of the primary results were not excessively altered by the omission of any individual study, confirming the robustness of the results.

### Heterogeneity and Publication Bias

No heterogeneity was observed. Several outcomes with a high *I*^2^ (>50%) and non-significant *P*-values for the Q statistic (>0.05) were still regarded as having low heterogeneity and were analyzed with a fixed-effects model (extratemporal or temporal location in ictal EEG patterns: *p* = 0.05, *I*^2^ = 53%; surgical strategy: *p* = 0.05, *I*^2^ = 53%). A random-effects model was used for age at infection (*p* = 0.02, *I*^2^ = 67%) and surgery (*p* = 0.006, *I*^2^ = 70%), time from seizure (*p* = 0.004, *I*^2^ = 74%) and infection (*p* = 0.002, *I*^2^ = 76%), and unilateral or bilateral ictal pattern on interictal EEG (*p* = 0.06, *I*^2^ = 71%).

There was no significant potential publication bias among all the combined results according to a funnel plot and Begg's and Egger's tests. Because the number of articles was limited, publication bias was not evaluated in the outcomes for interictal EEG.

## Discussion

In recent decades, the risk factors associated with surgical outcomes in postinfective epilepsy have varied among different infectious agents and different studies. The present meta-analysis identified the following five predictors, which were associated with favorable outcomes: abnormal MRI finding, meningitis, temporal location, and regional and unilateral ictal patterns. In addition, regional and unilateral ictal patterns were related to a good prognosis for viral encephalitis-related epilepsy surgery; however, we failed to identify any significant risk factors for other types of CNSI-related epilepsy.

There was no heterogeneity in most of the pooled results. Different outcome classification standards may influence the heterogeneity of combined results. Two studies conducted by Davies (23, 24) regarded seizure freedom as predictive of a favorable prognosis; however, this was not defined as the Engel classification (I/II). There was significant heterogeneity in age at the time of surgery and the time between seizure onset and infection.

The present study shows that a temporal location and abnormal MRI findings are predictors of a favorable outcome, which is consistent with the results obtained in other studies of focal cortical dysplasia (47, 48). There are many large adequate studies demonstrating that temporal lobectomies with structural abnormalities are the best patients for favorable outcome not only in patients with cortical dysplasia but with tumors especially developmental and mesial temporal sclerosis (MTS) (49). Meningitis is another protective factor that lowers the risk of epilepsy by 69% relative to the risk associated with encephalitis. Meningitis is a diffuse inflammatory change in the meninges. Compared with viral encephalitis, meningitis has less of an effect on neurons or connections between neurons, which may explain the above phenomenon. The differences between bacterial meningitis and viral encephalitis indicate an increased likelihood of a more extensive (less localized) impact of the latter, with a potential impact on the worst outcome. Moreover, patients with an older age at onset, a longer silent period, and a longer time from infection to seizure have more favorable surgical outcomes. In line with the results of previous studies of viral encephalitis-related epilepsy (38, 45, 50, 51), we found that a longer silent period between acute infection and the onset of epilepsy is a predictor of favorable postsurgical outcomes, although different types of infection did not produce significant effects in the subgroup analysis. Older age at onset may also reflect a longer silent period, which is a significant predictor of postsurgical outcomes. However, the time between surgery and encephalitis or meningitis is a novel factor that has not been reported in previous studies. A longer time from infection to one of these conditions suggests that the patient is a good candidate for epilepsy surgery, particularly in unexplained meningitis. Interestingly, better outcomes associated with patients with a history of meningitis, longer silent period, and temporal location are consistent with the diagnosis of secondary mesial temporal sclerosis, with well-known high odds of a good surgical outcome. Additionally, patients presenting with regional and unilateral ictal patterns have more favorable surgical outcomes than those with non-localizable and bilateral ictal patterns, which is consistent with a previous systematic review of focal cortical dysplasia (48). Non-localizable and bilateral ictal patterns may imply incomplete resection of the epileptogenic focus, which is likely to result in seizure recurrence.

The mechanisms by which different infectious agents produce acute seizures and then later on unprovoked seizures have not been fully addressed. The occurrence and development of epilepsy after brain infection vary with the type of infection, and it is often multifactorial. In meningitis and encephalitis, triggering of the inflammatory cascade seems to be a common potential factor that contributes to epileptogenesis. In patients with CNS infections, structural destruction and damage, such as infarction in meningitis, cortical necrosis with herpes simplex virus, hypoxic–ischemic injury in cerebral malaria, space-occupying effect of neurocysticercosis, and gliosis around calcified neurocysticercosis, might all form epileptogenic zones (3). Unfortunately, there are no published articles on the intergroup comparison of epilepsy susceptibility to bacterial meningitis, viral encephalitis, parasitic encephalopathy, and unexplained meningitis.

Potential limitations to this research should be acknowledged. First, all the studies used a retrospective design, and the results are, therefore, subject to selection and recall bias. Second, some of the studies we selected from the databases mainly focused on patients who achieved a seizure-free status instead of a favorable outcome according to the Engel classification. To include more potential studies in the present meta-analysis, we also accepted seizure freedom. Finally, unmeasured factors may have confounded the true association for unadjusted data used in the original studies.

Our meta-analysis also has some implications for future research. Few recent systematic reviews have focused on outcome data in postinfective epilepsy surgery, and different clinical studies of diverse infective agents have provided various predictors of prognoses (2). Therefore, the predictive factors identified here, including abnormal MRI findings, meningitis, temporal location only, regional and unilateral ictal patterns, older age at epilepsy, longer silent period, and longer time from infection, will be useful in the perioperative period. Based on a clinical diagnosis and identified predictors, doctors will be able to choose effective surgical procedures, determine nursing methods, and design dosage regimens in patients with a potentially high recurrence rate.

In conclusion, this meta-analysis identified eight factors, including abnormal MRI findings, meningitis, temporal location only, regional and unilateral ictal patterns, older age at epilepsy onset, longer silent period, and longer time from infection, that act as favorable predictors of surgical outcomes in CNSI-related epilepsy. In viral encephalitis-related epilepsy, regional and unilateral ictal patterns predicted favorable surgical outcomes. Additionally, a longer time from onset and a longer time from infection for unexplained meningitis were predictors of favorable surgical outcomes.

## Data Availability Statement

The original contributions presented in the study are included in the article/Supplementary Material, further inquiries can be directed to the corresponding author/s.

## Author Contributions

ZH: designing the study, searching and screening the literature, extracting and analyzing the data, and drafting the manuscript. NA, HY, and S-YL: collating the data. C-QZ: designing study and guiding manuscript writing. Q-TD: revising the manuscript. Y-YK: processing the data sets and manuscript revision. All authors read and approved the final manuscript.

## Conflict of Interest

The authors declare that the research was conducted in the absence of any commercial or financial relationships that could be construed as a potential conflict of interest.

## Publisher's Note

All claims expressed in this article are solely those of the authors and do not necessarily represent those of their affiliated organizations, or those of the publisher, the editors and the reviewers. Any product that may be evaluated in this article, or claim that may be made by its manufacturer, is not guaranteed or endorsed by the publisher.
