# Supplementary material for: Predictors of Seizure Freedom in Patients Undergoing Surgery for Central Nervous System Infection-Related Epilepsy: A Systematic Review and Meta-Analysis
Source: Front Neurol. 2021 Aug 18;12:668439. doi: 10.3389/fneur.2021.668439 (PMC8416488; doi:10.3389/fneur.2021.668439)
Supplement: Supplementary file 1 [file Data_Sheet_1.docx]

Search Strategy

PubMed

("Central Nervous System Infections"[Mesh] OR "Central Nervous System Infections"[All Fields] OR "Viral encephalitis"[All Fields]) AND ("Epilepsy"[Mesh] OR "Epilepsy"[All Fields]) AND ("Surgical Procedures, Operative"[Mesh] OR "Surgery"[All Fields] OR "Procedure"[All Fields] OR "Operation"[All Fields])

EMBASE

('central nervous system infections'/exp OR 'central nervous system infections' OR 'viral encephalitis'/exp OR 'viral encephalitis') AND ('epilepsy'/exp OR 'epilepsy') AND ('surgical procedures, operative'/exp OR 'surgery'/exp OR 'surgery' OR 'procedure'/exp OR 'procedure' OR 'operation'/exp OR 'operation') AND [humans]/lim AND [clinical study]/lim AND ([article]/lim OR [article in press]/lim OR [conference abstract]/lim OR [conference paper]/lim OR [letter]/lim OR [short survey]/lim) NOT ('case report'/exp OR 'case report' OR 'animal experiment'/exp OR 'animal experiment' OR 'review'/exp OR 'review' OR 'systematic review'/exp OR 'systematic review' OR 'practice guideline'/exp OR 'practice guideline')

Cochrane Library

#1 MeSH descriptor: [Central Nervous System Infections] explode all trees 1058

#2 Central Nervous System Infections 976

#3 Viral encephalitis 227

#4 MeSH descriptor: [Epilepsy] explode all trees 2400

#5 Epilepsy 6516

#6 MeSH descriptor: [Surgical Procedures, Operative] explode all trees 122520

#7 Surgery 174826

#8 Procedure 131547

#9 Operation 25720

#10 (#1 or #2 or #3) and (#4 or #5) and (#6 or #7 or #8 or #9) 87

CBM

("中枢神经系统感染"[主题词] OR "中枢神经系统感染"[常用字段] OR "病毒性脑炎"[常用字段]) AND ("癫痫"[主题词] OR "癫痫"[常用字段]) AND ("外科手术"[主题词] OR "外科"[常用字段] OR "手术"[常用字段])

CNKI

SU=(('中枢神经系统感染'+'病毒性脑炎')*'癫痫'*('外科手术'+'外科'+'手术'))

VIP

(m=中枢神经系统感染+m=病毒性脑炎)*m=癫痫*(m=外科手术+m=外科+m=手术)

WANFANG

主题:((中枢神经系统感染 OR 病毒性脑炎) AND 癫痫 AND (外科手术 OR 外科 OR 手术))
